# Long-Term Outcomes in Patients With Type 2 Myocardial Infarction and Myocardial Injury

**DOI:** 10.1161/CIRCULATIONAHA.117.031806

**Published:** 2018-03-12

**Authors:** Andrew R. Chapman, Anoop S.V. Shah, Kuan Ken Lee, Atul Anand, Oliver Francis, Philip Adamson, David A. McAllister, Fiona E. Strachan, David E. Newby, Nicholas L. Mills

**Affiliations:** 1 1 British Heart Foundation, University Centre for Cardiovascular Science, University of Edinburgh, United Kingdom (A.R.C., A.S.V.S., K.K.L., A.A., O.F., P.A., F.E.S., D.E.N., N.L.M.); 2 2 Institute for Health and Wellbeing, University of Glasgow, United Kingdom (D.A.M.)

**Keywords:** myocardial injury, troponin, type 2 myocardial infarction

## Abstract

**Background:**

Type 2 myocardial infarction and myocardial injury are common in clinical practice, but long-term consequences are uncertain. We aimed to define long-term outcomes and explore risk stratification in patients with type 2 myocardial infarction and myocardial injury.

**Methods:**

We identified consecutive patients (n=2122) with elevated cardiac troponin I concentrations (≥0.05 µg/L) at a tertiary cardiac center. All diagnoses were adjudicated as per the universal definition of myocardial infarction. The primary outcome was all-cause death. Secondary outcomes included major adverse cardiovascular events (eg, nonfatal myocardial infarction or cardiovascular death) and noncardiovascular death. To explore competing risks, cause-specific hazard ratios were obtained using Cox regression models.

**Results:**

The adjudicated index diagnosis was type 1 or 2 myocardial infarction or myocardial injury in 1171 (55.2%), 429 (20.2%), and 522 (24.6%) patients, respectively. At 5 years, all-cause death rates were higher in those with type 2 myocardial infarction (62.5%) or myocardial injury (72.4%) compared with type 1 myocardial infarction (36.7%). The majority of excess deaths in those with type 2 myocardial infarction or myocardial injury were because of noncardiovascular causes (hazard ratio, 2.32; 95% confidence interval, 1.92–2.81 versus type 1 myocardial infarction). Despite this finding, the observed crude major adverse cardiovascular event rates were similar between groups (30.6% versus 32.6%), with differences apparent after adjustment for covariates (hazard ratio, 0.82; 95% confidence interval, 0.69–0.96). Coronary heart disease was an independent predictor of major adverse cardiovascular events in those with type 2 myocardial infarction or myocardial injury (hazard ratio, 1.71; 95% confidence interval, 1.31–2.24).

**Conclusions:**

Despite an excess in noncardiovascular death, patients with type 2 myocardial infarction or myocardial injury have a similar crude rate of major adverse cardiovascular events as those with type 1 myocardial infarction. Identifying underlying coronary heart disease in this vulnerable population may help target therapies that could modify future risk.

## Abstract 2

Supplemental Digital Content is available in the text.

**Clinical Perspective**

**What Is New?**

- We report long-term outcomes at 5 years in consecutive patients with type 1 or type 2 myocardial infarction or myocardial injury.
- Two-thirds of patients with type 2 myocardial infarction or myocardial injury are dead at 5 years, with a similar rate of future nonfatal myocardial infarction or cardiovascular death as those with type 1 myocardial infarction.
- The presence of coronary artery disease is an independent predictor of future cardiovascular risk in patients with type 2 myocardial infarction or myocardial injury.

**What Are the Clinical Implications?**

- Clinicians should consider risk stratification in patients with type 2 myocardial infarction or myocardial injury for the likelihood of coronary artery disease.
- Prospective clinical trials are needed to define the efficacy and safety of secondary prevention therapies in patients with type 2 myocardial infarction or myocardial injury, which have the potential to modify future outcomes.

The diagnostic criteria for acute myocardial infarction were updated to accommodate the introduction of more sensitive cardiac troponin assays and in recognition of the wide range of conditions associated with myocardial injury.^1^ The third universal definition of myocardial infarction recommends a classification that is based on etiology, where type 1 myocardial infarction is because of plaque rupture or erosion with atherothrombotic consequences and type 2 myocardial infarction because of myocardial oxygen supply–demand imbalance in the absence of atherothrombosis. Patients with elevated cardiac troponin concentrations who do not have overt myocardial ischemia are classified as having myocardial injury.^2^ Although these diagnostic categories are considered distinct in guidelines, implementation in clinical practice has been challenging because of similarities between patients with type 2 myocardial infarction and myocardial injury, with the implications of these diagnoses uncertain.

The Global Task Force is reviewing the classification of myocardial infarction and recognizes the need to provide greater clarity for clinicians in practice.^3^ Although patients with type 2 myocardial infarction and myocardial injury have higher crude rates of all-cause death compared with those with type 1 myocardial infarction,^4–9^ differences do not always persist in adjusted analyses,^10,11^ and few studies report cause of death or risk of future cardiovascular events.^12^ If patients with type 2 myocardial infarction are at increased risk of cardiovascular events attributable to atherosclerotic disease, then targeted investigation and preventative therapies have the potential to modify outcomes.

In consecutive patients with elevated cardiac troponin concentrations measured using a sensitive assay, we previously observed that the diagnosis of type 2 myocardial infarction or myocardial injury was as common as type 1 myocardial infarction.^4^ Here we report outcomes for these patients and determine the clinical features associated with major adverse cardiovascular events, with the aim of improving risk stratification in patients with type 2 myocardial infarction or myocardial injury.

## Methods

### Transparency and Openness Promotion

The analysis code for this study has been made available online (Appendix I in the online-only Data Supplement). The data will not be made available to other researchers for the purposes of reproducing the results because of lack of data sharing approval.

### Study Population

Consecutive hospital inpatients with elevated cardiac troponin I concentrations (≥0.05 µg/L) were identified at a tertiary cardiac center (Royal Infirmary of Edinburgh, Scotland, United Kingdom) during the validation (January 19, 2008–July 31, 2008) and implementation (January 19,2009–July 31, 2009) phases of a contemporary sensitive cardiac troponin I assay.^4,13^ We included all patients in whom cardiac troponin was requested by the attending clinician regardless of suspected etiology or hospital department. All clinical details were obtained using an electronic patient record (TrakCare, InterSystems). We excluded patients admitted for elective procedures, those with incomplete electronic hospital records, and patients who were not residents to ensure complete follow-up.

### Cardiac Troponin Assay

Plasma cardiac troponin concentrations were measured using a contemporary sensitive cardiac troponin I assay (ARCHITECT_*STAT*_, Abbott Laboratories, Abbott Park, IL). The study was divided into validation and implementation phases.^4,13^ Only cardiac troponin concentrations above the diagnostic threshold of the previous generation assay (≥0.20 µg/L) were reported to clinicians during the validation phase, whereas concentrations above a revised diagnostic threshold (≥0.05 µg/L) were reported during the implementation phase. The 99th percentile of this assay is 0.028 µg/L; however, a diagnostic threshold of ≥0.05 µg/L was implemented because this was the minimum concentration where the coefficient of variation was <10% under local laboratory conditions. All troponin results were available to the research team irrespective of study phase.

### Diagnostic Classification

All diagnoses were classified as per the 3rd universal definition of myocardial infarction.^2,4^ Patients were classified as having a type 1 myocardial infarction when myocardial necrosis occurred in the context of a presentation with suspected acute coronary syndrome with symptoms of myocardial ischemia or evidence of myocardial ischemia on the electrocardiogram. Patients with symptoms or signs of myocardial ischemia that were thought to be because of increased oxygen demand (eg, tachyarrhythmia or hypertrophy) or decreased supply (eg, hypotension, hypoxia, or anemia) and myocardial necrosis in the context of an alternative clinical diagnosis were classified as having a type 2 myocardial infarction. Myocardial injury was defined as evidence of myocardial necrosis in the absence of any symptoms or signs of myocardial ischemia. For this analysis, we excluded patients classified as having type 3, type 4a, type 4b, or type 5 myocardial infarction. Each case was reviewed and classified independently by 2 cardiologists, and any discrepancies were resolved by consensus through in-depth review of source data. Further information on the adjudication process is provided in Appendix II in the online-only Data Supplement.

### Clinical Outcomes

Clinical outcomes were identified using local and national population registries. We determined death using TrakCare (InterSystems) and the National Register of Scotland, with future hospitalization for myocardial infarction or heart failure identified using an extract from the Scottish Morbidity Record. We defined death from a cardiovascular cause where 1 of the following International Classification of Diseases-10 codes was listed as the primary cause of death: I20–25, I34–37, I42–43, I46, I48–51, and I60–69 (Appendix III in the online-only Data Supplement). The primary outcome was all-cause death. Secondary outcomes included major adverse cardiovascular events (MACEs; defined as cardiovascular death or subsequent myocardial infarction), nonfatal myocardial infarction, fatal myocardial infarction, hospitalization with heart failure, and noncardiovascular death. We obtained follow-up for all patients until the primary outcome or date of censoring (November 16, 2015).

### Ethical Considerations

The parent study protocol evaluated the implementation of a sensitive cardiac troponin assay and was deemed to fall under the remit of audit and service evaluation by the National Health Service Lothian Regional Ethics Committee, and therefore formal ethical approval was not required. For this study, we received approval from the Caldicott guardian to obtain long-term follow-up through local and national registries.

### Statistical Analysis

Baseline characteristics were summarized as mean (standard deviation) or median (interquartile range) as appropriate, with patients grouped on the basis of the classification of myocardial infarction. Crude incidence rates for primary and secondary outcomes were calculated, with risk ratios obtained using a generalized linear model with a log link, Poisson error distribution, and robust variance estimates.^14^ We adjusted for clinically relevant covariates, including age, sex, renal function (estimated glomerular filtration rate), hemoglobin (g/L), diabetes mellitus, hypertension, coronary heart disease (defined as previous myocardial infarction, coronary revascularization, or known angina pectoris), stroke, peripheral vascular disease, or cigarette smoking. The study period included a lowering of the upper reference limit for cardiac troponin from 0.20 µg/L (validation phase) to 0.05 µg/L (implementation phase), and we therefore included a study phase in all models. We repeated these analyses among only those patients who survived 30 days after presentation, defining the start of the follow-up period as 30 days after presentation. To explore competing risks, cause-specific hazard ratios were obtained using Cox regression models for type 1 myocardial infarction versus type 2 myocardial infarction or myocardial injury for MACE and noncardiovascular death. Penalized splines were used to accommodate departures from linearity. We examined for nonproportional hazards graphically and via the method proposed by Grambsch and Therneau.^15^ In patients who survived to 30 days, we explored associations between covariates and future risk of MACE. Cumulative incidence plots were produced for secondary cardiovascular outcomes, which also illustrate the competing risk of noncardiovascular death. We report 95% confidence intervals (CIs) for all estimates, with all analyses performed using R (version 3.2.2) using the *survival* and *cmprsk* packages.^16^

## Results

We identified 2929 consecutive patients with elevated cardiac troponin concentrations (≥0.05 µg/L) of whom 807 met our exclusion criteria (Figure I in the online-only Data Supplement). In the study population (n=2122), the adjudicated diagnosis was type 1 myocardial infarction in 1171 patients (55.2%), type 2 myocardial infarction in 429 patients (20.2%), and myocardial injury in 522 patients (24.6%; Table 1).

**Table 1.**
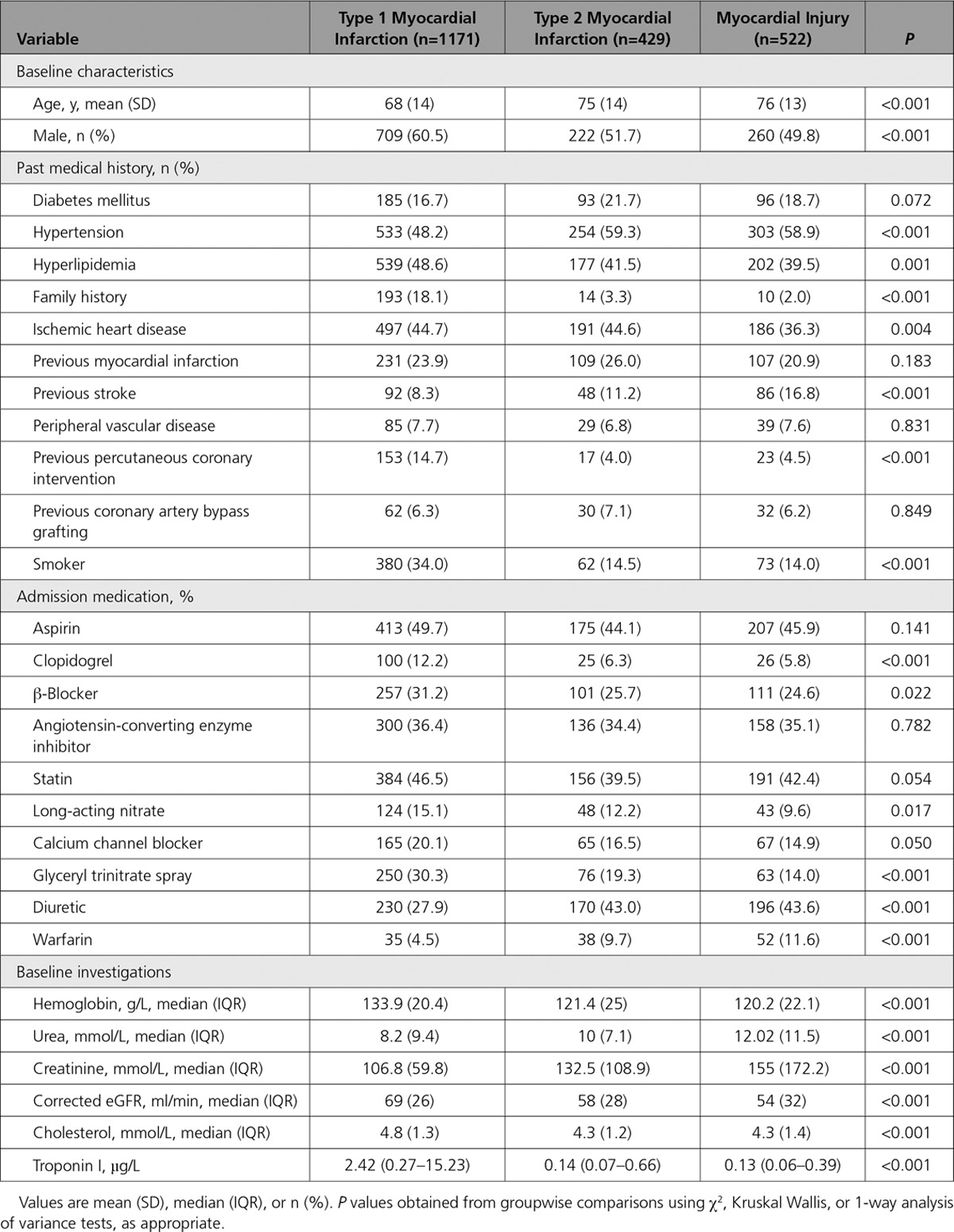
Baseline Characteristics of the Study Population

### Clinical Characteristics

Patients with type 2 myocardial infarction or myocardial injury were older, and there was a higher proportion of women than men compared with patients with type 1 myocardial infarction. Anemia or renal impairment was more common in patients with type 2 myocardial infarction or myocardial injury. A history of previous coronary revascularization was more frequent in those with type 1 myocardial infarction. At presentation, the prescription of antiplatelet, antihypertensive, and lipid-lowering therapies was similar across all patients (Table 1). The most common diagnoses in patients with type 2 myocardial infarction or myocardial injury were cardiac arrhythmia, decompensated left ventricular failure, pneumonia, or long bone fracture, with variation in prevalence by classification (Table I in the online-only Data Supplement).

### Clinical Outcomes at 5 Years in All Patients

**Figure 1.**
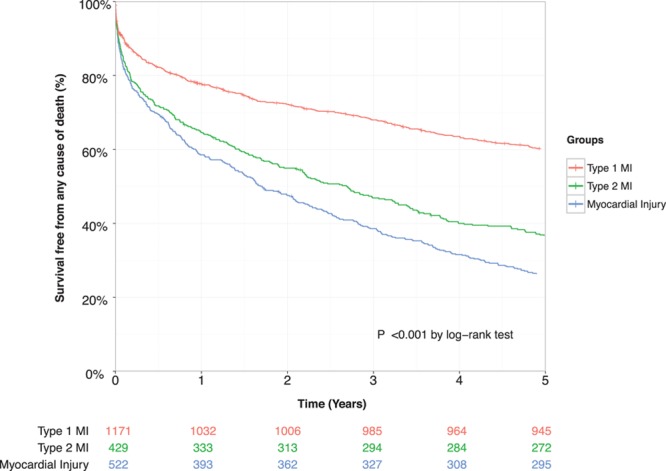
**Kaplan–Meier curves illustrating risk of death from any cause at 5 years stratified by index diagnosis, with table of number at risk.** Pairwise comparison of groups obtained using the log-rank test. MI indicates myocardial infarction.

During 8809 person-years follow-up (median 4.9 years), death from any cause occurred in 1231 patients (58%). In patients with type 2 myocardial infarction, at 5 years, the observed risk of death was higher compared with those with type 1 myocardial infarction (62.5% versus 36.7%; unadjusted relative risk [RR], 2.15; 95% CI, 1.82–2.55). After incorporating age, sex, renal function, hemoglobin, and other clinically relevant covariates, the adjusted RR fell to 1.51 (95% CI, 1.21–1.87) (Table 2, Figure 1).

**Table 2.**
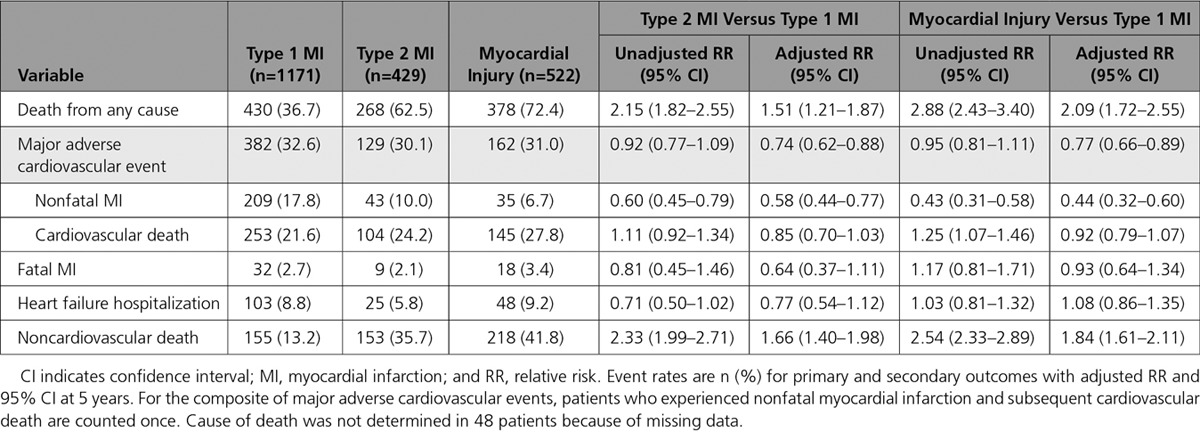
Death and Major Cardiovascular Events at 5 Years, by Diagnosis

**Figure 2.**
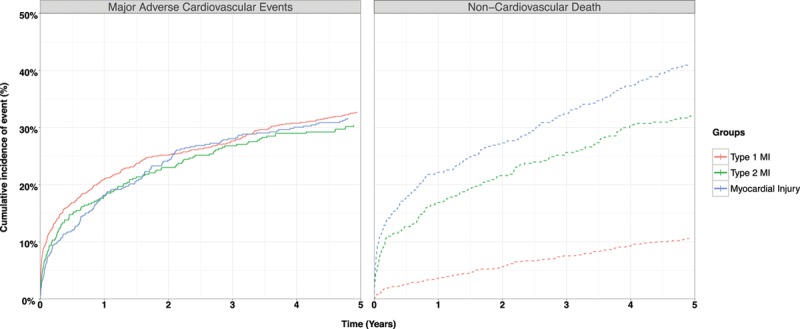
**Cumulative incidence curves illustrating risk of major adverse cardiovascular events (type 1 myocardial infarction [MI] or cardiovascular death) and competing risk of noncardiovascular death at 5 years stratified by index diagnosis.**

The 5-year risk of nonfatal myocardial infarction or cardiovascular death (MACE) was similar in patients with type 2 compared with type 1 myocardial infarction (30.1% versus 32.6%; unadjusted RR, 0.92; 95% CI, 0.77–1.09) (Figure 2) but lower after adjustment for age, sex, and other covariates (adjusted RR, 0.74; 95% CI, 0.62–0.88). Adjusting for the same covariates, the cause-specific HR for MACEs (with noncardiovascular mortality as the competing outcome) was similar to the RR (HR, 0.82; 95% CI, 0.69–0.96) (Table 3, Table II in the online-only Data Supplement).

**Table 3.**
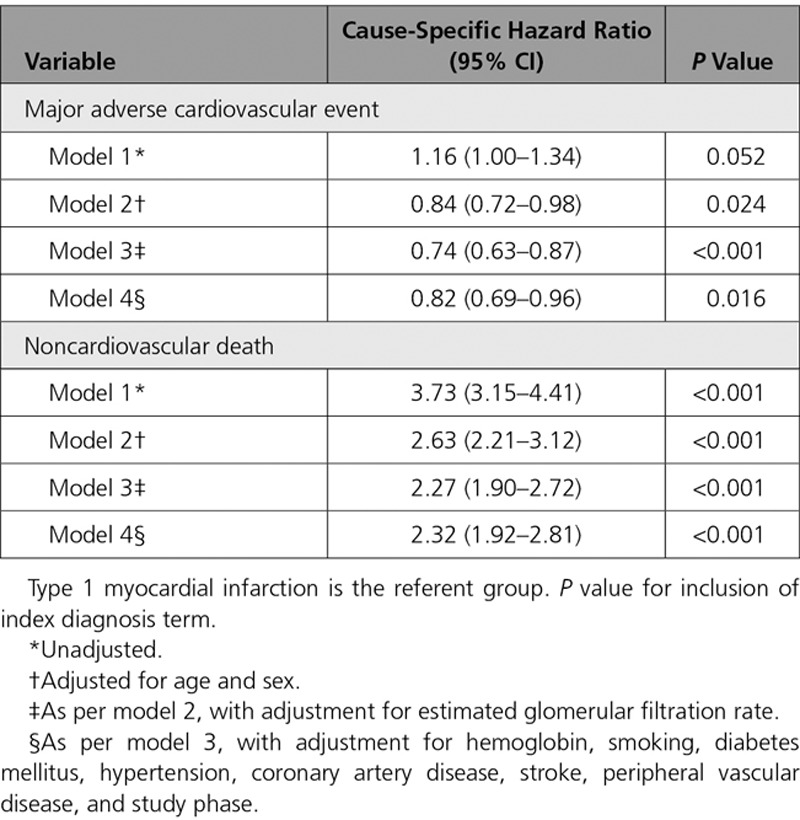
Cause-Specific Hazard Ratio for Major Adverse Cardiovascular Event and Noncardiovascular Death in Patients With Type 2 Myocardial Infarction or Myocardial Injury Versus Type 1 Myocardial Infarction in Unadjusted and Fully Adjusted Cox-Regression Models

For the individual components of MACEs, the risk of nonfatal myocardial infarction was lower in those with type 2 myocardial infarction compared with type 1 myocardial infarction (10.0% versus 17.8%; adjusted RR, 0.58; 95% CI, 0.44–0.77). Although the crude rates of cardiovascular death were higher for type 2 myocardial infarction compared with type 1 myocardial infarction (24.2% versus 21.6%), the adjusted relative risk was lower at 0.85 (95% CI, 0.70–1.03). Risks of fatal myocardial infarction and hospitalization with heart failure were comparable across groups (Table 2). Noncardiovascular death was higher in patients with type 2 myocardial infarction compared with type 1 myocardial infarction (35.7% versus 13.2%; adjusted RR, 1.66; 95% CI, 1.40–1.98) (Figure 2).

We found similar relative risks for patients with myocardial injury compared with type 1 myocardial infarction for most primary and secondary outcomes, but a lower risk of nonfatal myocardial infarction and higher risk of noncardiovascular death were observed. Patients with myocardial injury had a higher risk of all-cause death and heart failure hospitalization than patients with type 2 myocardial infarction (Table III in the online-only Data Supplement).

### Clinical Outcomes at 5 Years in Those Who Survive to 30 Days

In patients who survived from their initial presentation to 30 days, death from any cause occurred in 31% (333/1074) of patients with type 1 myocardial infarction, 56.1% (207/368) of patients with type 2 myocardial infarction, and 67% (293/437) of patients with myocardial injury (Table IV in the online-only Data Supplement). The adjusted RR of death for patients with type 2 versus type 1 myocardial infarction was similar to that observed in the total population (adjusted RR, 1.52; 95% CI, 1.21–1.92). For all but 1 of the secondary outcomes, the relative risks were similar to those obtained in the main analysis. However, the association between type of myocardial infarction and risk of MACE was weaker than was observed in the whole population, occurring in 27.4% (101/368) of patients with type 2 myocardial infarction and 27.7% (298/1074) of patients with type 1 myocardial infarction, with an adjusted RR of 0.80 (95% CI, 0.65–0.98).

**Figure 3.**
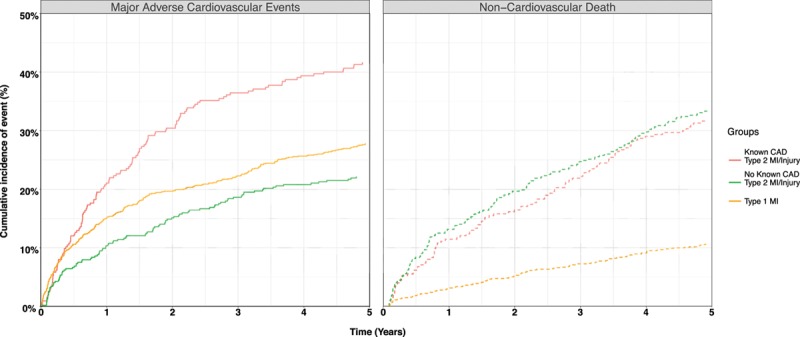
**Cumulative incidence curves illustrating risk of major adverse cardiovascular events (type 1 myocardial infarction or cardiovascular death) and competing risk of noncardiovascular death in those who survive to 30 days in patients with type 1 myocardial infarction and in those with type 2 myocardial infarction or myocardial injury stratified by known CAD.** CAD indicates coronary artery disease; and MI, myocardial infarction.

In patients with type 2 myocardial infarction or myocardial injury, age, declining renal function, a history of diabetes mellitus, peripheral vascular disease, and coronary artery disease were independent predictors of MACEs at 5 years (Table V in the online-only Data Supplement). The presence of coronary artery disease was associated with an increase in the cause-specific hazard ratio for MACEs at 5 years (HR, 1.71; 95% CI, 1.31–2.24) compared with those without coronary artery disease. When compared with patients with type 1 myocardial infarction, patients with type 2 myocardial infarction or myocardial injury with coronary artery disease had a higher risk of a MACE (RR, 1.56; 95% CI, 1.29–1.88). The adjusted cause-specific hazard ratio for MACE, which accounts for competing risk from noncardiovascular death, was 1.05 (95% CI, 0.85–1.30) (Figure 3). On discharge from hospital, patients with type 2 myocardial infarction or myocardial injury and a history of coronary artery disease were less likely than those with type 1 myocardial infarction to be prescribed aspirin (66.2% versus 90.7%), a statin (69.2% versus 86.0%), or an angiotensin converting enzyme inhibitor (52.9% versus 71.3%, *P*<0.001 for all) (Table 4).

**Table 4.**
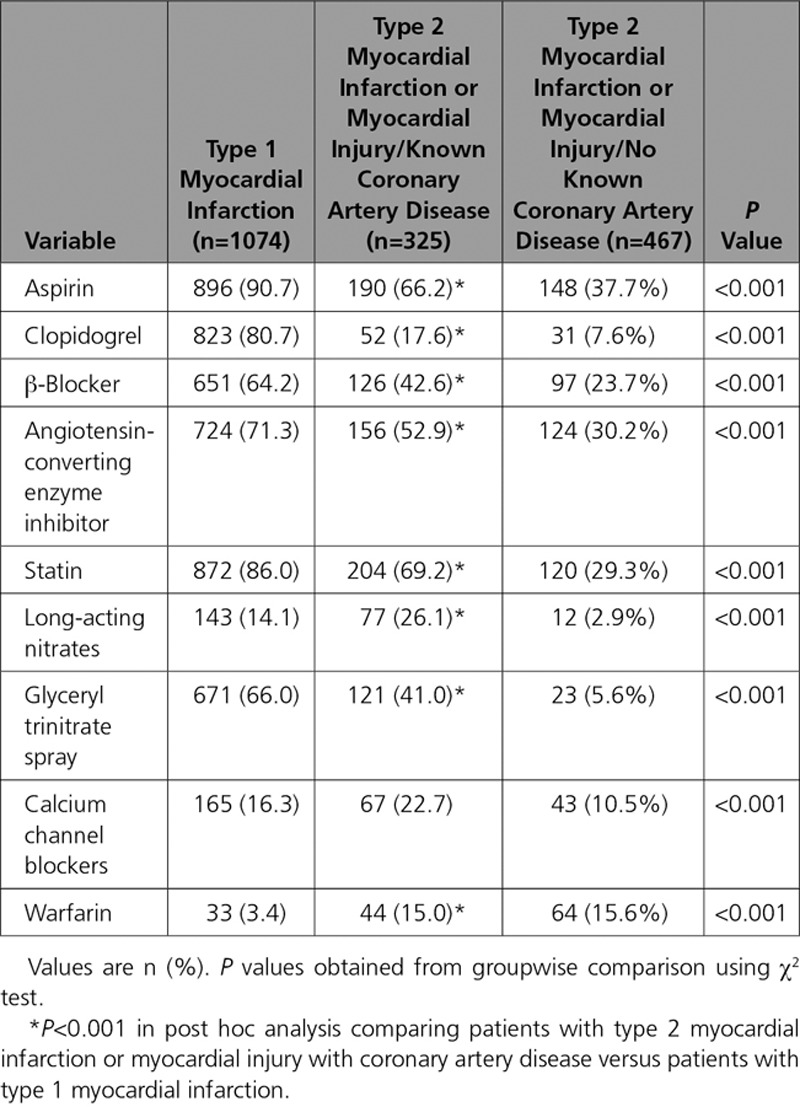
Recommended Therapies at Discharge in Patients With Type 1 Myocardial Infarction, Type 2 Myocardial Infarction, and Myocardial Injury Who Survive to 30 Days, Stratified by the Presence of Coronary Artery Disease

## Discussion

In a cohort of consecutive hospitalized patients with elevated cardiac troponin concentrations, we classified the diagnosis of myocardial infarction according to the universal definition and report outcomes after 5 years follow-up. We make several observations that have implications for clinical practice. First, greater than two thirds of patients with type 2 myocardial infarction or myocardial injury do not survive to 5 years after index diagnosis. This mortality rate was twice that of patients with type 1 myocardial infarction, with differences primarily because of an excess in noncardiovascular deaths. Second, MACEs occurred in one third of patients, and rates were similar irrespective of diagnostic classification. In those patients with type 2 myocardial infarction or myocardial injury, the presence of coronary heart disease was 1 of the strongest predictors of a MACE. Those patients with type 2 myocardial infarction or myocardial injury with known coronary artery disease were less likely to receive secondary prevention therapies compared with those with type 1 myocardial infarction. Identifying patients with elevated cardiac troponin concentrations in the context of an acute illness who have underlying coronary heart disease may provide an opportunity for clinicians to improve the targeting of preventative therapies and reduce the risk of cardiovascular events.

Several studies demonstrate that the diagnosis of type 2 myocardial infarction is common in clinical practice. It is responsible for between 2% and 37% of all elevations in cardiac troponin in unselected hospitalized patients and between 5% to 71% in unselected patients attending the emergency department.^17–21^ Myocardial injury has been reported in ≤70% of unselected patients,^5,22^ but because the frequency of diagnosis is not reported by the majority of studies, failure to classify patients according to the criteria set out in the universal definition may inflate the incidence of type 2 myocardial infarction.^23^ Both type 2 myocardial infarction and myocardial injury increase the risk of all-cause death at ≤3 years.^5–9,21,23–25^ We now provide outcome data at 5 years demonstrating that two thirds of patients with type 2 myocardial infarction or myocardial injury are dead with twice the event rate of patients with type 1 myocardial infarction.

One of the key limitations of prior analyses is that the majority have not reported the specific cause of death, and therefore estimates of the proportion of events that may be attributable to cardiovascular disease are lacking.^26,27^ We found that the excess in all-cause death in patients with type 2 myocardial infarction or myocardial injury was largely attributable to a 3-fold increase in noncardiovascular death. Because patients with type 2 myocardial infarction or myocardial injury are older and have a higher prevalence of anemia, renal impairment, and other comorbidities, this outcome is perhaps unsurprising. Nonetheless, it is notable that the crude risk of MACEs in patients with type 2 myocardial infarction or myocardial injury was similar to that in patients with type 1 myocardial infarction. In models taking into account the differences in age, sex, and other characteristics between patients with different index diagnoses, the risk of subsequent cardiovascular events was ≈25% lower in patients with type 2 myocardial infarction or myocardial injury than in patients with type 1 myocardial infarction. This may in part be attributable to competing risks, with the much higher rates of noncardiovascular death reducing the pool of patients at risk of having a cardiovascular event. However, competing risks are not the only explanation for the lower rates of MACE in patients with type 2 myocardial infarction or myocardial injury, as in an adjusted analysis taking into account competing risks and other clinical variables, a difference in the cause-specific hazard ratio was still apparent between the groups.

The diagnostic distinction between patients with type 2 myocardial infarction and myocardial injury is challenging but worthwhile if the diagnosis conveys important prognostic information or influences treatment decisions.^7,28–30^ In our analysis, the recommended classification of type 2 myocardial infarction or myocardial injury did not differentially identify those patients at risk of a MACE. This observation is consistent with previous studies and suggests that alternate strategies for risk stratification may be required. In patients with type 2 myocardial infarction, the presence of obstructive coronary artery disease may influence prognosis. Outcomes from the SWEDEHEART registry (Swedish Web-system for Enhancement and Development of Evidence-based care in Heart disease Evaluated According to Recommended Therapies) of 41 817 patients with type 1 or 2 myocardial infarction demonstrated an increased risk of all-cause death in patients with type 2 myocardial infarction with obstructive coronary artery disease compared with those without.^21^ Similarly, in a recent analysis of the APACE cohort (Advantageous Predictors of Acute Coronary Syndromes Evaluation), Nestelberger et al^31^ found that patients with type 2 myocardial infarction and coronary artery disease had a 90-day cardiovascular mortality of 3.6%, with no deaths observed in those without coronary artery disease. Our analysis supports these findings, with coronary artery disease 1 of the strongest predictors of MACEs in patients with type 2 myocardial infarction or myocardial injury. The prevalence of coronary artery disease in patients with type 2 myocardial infarction or myocardial injury was 42% in our cohort and varies between 36% and 78% in previous reports.^7,11,21,22,32^ However, estimates obtained from registry studies are hindered by selection bias because those who undergo angiography will have a higher pretest probability of coronary artery disease, therefore the true prevalence of coronary artery disease in this group of patients remains uncertain.^33^

It is important to note that patients with type 2 myocardial infarction or myocardial injury receive fewer prescriptions for preventative therapies compared with those with type 1 myocardial infarction.^9,10,20–23^ To date, no randomized controlled trials have evaluated secondary prevention in this population, and there are no formal recommendations for risk assessment or treatment.^30^ Given the current heterogeneity in application of the universal definition of myocardial infarction, the feasibility of delivering such a study with comparable observations across multiple healthcare settings is uncertain. Primary prevention guidelines recommend statin therapy where the predicted 10-year risk of adverse cardiovascular events is >10%.^34^ In our study, for patients who survive their initial presentation with type 2 myocardial infarction and are not already known to have coronary artery disease, the rate of MACEs is >10% at 1 year. Although this outcome may be partially attributable to age and the presence of comorbidities, a significant proportion may have unrecognized coronary artery disease and may benefit from further investigation or preventative therapies.

We believe that clinicians should adopt a pragmatic approach and risk stratify individual patients on the basis of their likelihood of coronary artery disease.^29,30^ There are no risk assessment tools validated for use in this setting, therefore clinicians must review the presenting symptoms, medical history, cardiovascular risk factors, serial 12-lead electrocardiograms, and any available imaging findings and apply clinical judgment. Where the probability of coronary disease is high, it may be reasonable to commence secondary prevention with aspirin and a statin in the absence of contraindications. If patients with type 2 myocardial infarction are found to have obstructive coronary artery disease, revascularization could plausibly reduce the risk of future cardiac events, but this strategy has not been evaluated. Where the probability of coronary disease is intermediate or low, further investigation (invasive or CT coronary angiography) should be considered to identify patients with underlying coronary artery disease, where the benefits of secondary prevention are well recognized. The optimal timing for investigation in this group of patients is also uncertain. Where the probability of type 1 myocardial infarction is high, invasive assessment should be considered on an urgent basis in line with standard practice. In those patients where myocardial injury or infarction is secondary to oxygen supply–demand imbalance, further assessment may need to be deferred until patients have recovered from their primary illness. Furthermore, a recognition that these patients are at increased risk of noncardiovascular events may lead to an improvement in outcomes, through better monitoring or intensification of treatment of the primary presenting condition.

There are important limitations to the data presented. The study population was identified on the basis of an elevated troponin I concentration measured using a contemporary sensitive assay with a diagnostic threshold of 0.05 µg/L, and the true prevalence of myocardial injury and infarction could be higher using a lower threshold or a high-sensitivity cardiac troponin assay. Although 2 cardiologists adjudicated index diagnoses using all available clinical information, with excellent intraobserver agreement, there remains potential for misclassification, particularly for type 2 myocardial infarction and myocardial injury. There is likely to be variation in the in-hospital treatments received, which we could not adjust for, and we could not adjust for illness severity. As previously reported, a low proportion of patients with type 2 myocardial infarction or myocardial injury underwent inpatient coronary angiography.^4^ We therefore defined coronary artery disease on the basis of a diagnosis of angina, previous myocardial infarction, or previous coronary revascularization, which is likely to significantly underestimate the prevalence of coronary artery disease. Last, subsequent hospitalizations and cardiovascular or noncardiovascular death were determined using International Classification of Diseases-10 coding obtained from regional and national registry data, where there is the potential for both diagnostic and coding errors. We were therefore not able to determine the incidence of subsequent type 1 or type 2 myocardial infarction.

## Conclusions

More than two thirds of patients admitted to hospital with type 2 myocardial infarction or myocardial injury die in ≤5 years, with the majority of deaths because of noncardiovascular causes. Nonetheless, MACEs occur in one third of patients with elevated cardiac troponin concentrations, irrespective of whether myocardial necrosis was spontaneous or secondary to another acute illness. Although patients with type 1 myocardial infarction were at highest risk, there was no separation of risk between those with a diagnosis of type 2 myocardial infarction or myocardial injury. In contrast, those patients with type 2 myocardial infarction or myocardial injury known to have coronary artery disease are at highest risk of cardiovascular events, and efforts to diagnose coronary artery disease may provide opportunities to target preventative therapies and improve patient outcomes.

## Sources of Funding

This work was supported by the British Heart Foundation (SP/12/10/29922 and PG/15/51/31596). Drs Chapman, Mills, and Newby are supported by a Clinical Research Training Fellowship (FS/16/75/32533), a Butler Senior Clinical Research Fellowship (FS/16/14/32023), and Chair (CH/09/002) awards from the British Heart Foundation. Dr McAllister is supported by an intermediate clinical fellowship from the Wellcome Trust (201492-Z-16-Z). Dr Anand is supported by a research fellowship from Chest Heart and Stroke Scotland (15/A163). Dr Newby is the recipient of a Wellcome Trust Senior Investigator Award (WT103782AIA).

## Disclosures

Drs Anand and Shah have received honoraria from Abbott Diagnostics. Dr Chapman has received honoraria from Abbott Diagnostics and Astra-Zeneca. Dr Mils has acted as a consultant for Abbott Diagnostics, Beckman-Coulter, Roche, and Singulex. The other authors report no conflicts of interest. The funders had no role in the design or conduct of the study; the collection, analysis, and interpretation of data; or the preparation, review, or approval of the article.

## Supplementary Material
