# Supplementary material for: Long-Term Outcomes in Patients With Type 2 Myocardial Infarction and Myocardial Injury
Source: Circulation. 2018 Mar 12;137(12):1236–45. doi: 10.1161/CIRCULATIONAHA.117.031806 (PMC5882250; doi:10.1161/CIRCULATIONAHA.117.031806)
Supplement: Supplementary file 1 [file cir-137-1236-s001.pdf]

SUPPLEMENTAL MATERIAL

Long term outcomes in patients with type 2 myocardial infarction and myocardial injury

Andrew R Chapman MD,[1] Anoop SV Shah MD PhD,[1] Kuan Ken Lee MD,[1]
Atul Anand MD,[1] Oliver Francis MD,[1] Philip Adamson MD,[1] David A McAllister MD,[2]
Fiona Strachan PhD,[1] David E. Newby MD PhD,[1] Nicholas L Mills MD PhD[1]

[1]*BHF Centre for Cardiovascular Science, University of Edinburgh, Edinburgh, United Kingdom*
[2]*Institute for Health and Wellbeing, University of Glasgow, Glasgow, United Kingdom*

Running title: *Long term outcomes of type 2 myocardial infarction*

Address for correspondence:
Dr Andrew R Chapman
BHF/University Centre for Cardiovascular Science
Chancellor's Building
University of Edinburgh
Edinburgh EH16 4SB
United Kingdom
Tel: +44 131 242 6431
Fax: +44 131 242 6379
E-mail: a.r.chapman@ed.ac.uk
Twitter: @chapdoc1

Supplementary Tables: 5

Supplementary Figures: 1

Supplementary Appendix: 3

Support: British Heart Foundation Special Project Grant (SP/12/10/29922), Project Grant (PG/15/51/31596), Clinical Research Training Fellowship (FS/16/75/32533) and Senior Clinical Research Fellowship (FS/16/14/32023). Chest Heart and Stroke Scotland Research Fellowship (15/A163). Intermediate Clinical Fellowship from the Wellcome Trust (201492-Z-16-Z).

Supplemental Table 1. Most common primary discharge diagnoses in patients with an adjudicated diagnosis of type 2 myocardial infarction or myocardial injury.

Type 2 Myocardial Infarction	*Myocardial Injury*
Arrhythmia (19.1%, 82/429)	Heart Failure (12.8%, 67/522)
Pneumonia (13.5%, 58/429)	Arrhythmia (10.9%, 57/522)
Heart Failure (12.4%, 53/429)	Pneumonia (9.6%, 50/522)
Fracture (4.2%, 18/429)	Fracture (8.0%, 42/522)

Supplemental Table 2 – Cause-specific hazard ratios for major adverse cardiovascular events in all patients.

	Major Adverse Cardiovascular Events (MACE)	
	Unadjusted HR (95% CI)	***Adjusted HR (95% CI)***
**Age (per 10-year increase)**	1.60 (1.50-1.70)	-
**Sex (male)**	0.85 (0.73-0.98)	1.09 (0.93-1.28)
**Haemoglobin (per 10 g/L reduction)**	1.18 (1.14-1.21)	1.07 (1.03-1.11)
**eGFR (per 10 ml/min reduction)**	1.20 (1.17-1.24)	-
**Smoking**	0.66 (0.55-0.79)	1.26 (1.02-1.56)
**Diabetes Mellitus**	1.77 (1.49-2.10)	1.36 (1.14-1.64)
**Hypertension**	1.66 (1.42-1.93)	1.05 (0.89-1.24)
**Coronary Artery Disease**	2.52 (2.16-2.94)	1.80 (1.52-2.14)
**Stroke**	1.88 (1.53-2.31)	1.10 (0.89-1.38)
**Peripheral Vascular Disease**	2.07 (1.65-2.59)	1.45 (1.14-1.86)
**Validation phase**	1.21 (1.04-1.40)	1.16 (0.99-1.35)
**Type 1 Myocardial Infarction**	1.00	1.00
**Type 2 Myocardial Infarction / Myocardial Injury**	1.16 (1.00-1.34)	0.82 (0.69-0.96)

Penalised smoothing splines used for age and eGFR (estimated glomerular filtration rate) in multivariate model. Type 1 Myocardial Infarction as referent group.

Supplemental Table 3 – Adjusted relative risks of primary and secondary outcomes for patients with myocardial injury versus type 2 myocardial infarction

	**Myocardial Injury versus Type 2 MI**<br><br>**Adjusted RR (95% CI)**
Death from any cause	1.27 (1.08-1.48)
MACE	0.99 (0.87-1.13)
Non-fatal MI	0.80 (0.61-1.03)
Cardiovascular death	1.07 (0.94-1.22)
Fatal MI	1.18 (0.87-1.58)
Heart failure hospitalization	1.23 (1.03-1.46)
Non-cardiovascular death	1.12 (0.99-1.26)

Models adjusted for age, gender, renal function, haemoglobin and history of hypertension, stroke, peripheral vascular disease, diabetes mellitus, smoking, coronary artery disease and study phase.

Supplemental Table 4. Death and major cardiovascular events at 5 years stratified by diagnosis in those who survived index hospitalization

	Type 1 MI (n=1,074)	Type 2 MI (n=368)	Myocardial injury (n=437)	Type 2 MI versus Type 1 MI<br><br>Adjusted RR (95% CI)	Myocardial Injury versus Type 1 MI<br><br>Adjusted RR (95% CI)
Death from any cause	333 (31.0%)	207 (56.1%)	293 (67.0%)	1.52 (1.21-1.92)	1.95 (1.60-2.39)
MACE	298 (27.7%)	101 (27.4%)	135 (30.9%)	0.80 (0.65-0.98)	0.87 (0.73-1.02)
Non-fatal MI	198 (18.4%)	41 (11.1%)	34 (7.8%)	0.60 (0.45-0.81)	0.46 (0.34-0.64)
Cardiovascular death	172 (16.0%)	77 (20.9%)	118 (27.0%)	0.95 (0.76-1.18)	1.07 (0.90-1.27)
Fatal MI	32 (3.0%)	9 (2.4%)	17 (3.9%)	0.65 (0.38-1.14)	0.90 (0.61-1.31)
Heart failure hospitalization	92 (8.6%)	22 (6.0%)	39 (8.9%)	0.86 (0.58-1.26)	1.18 (0.91-1.52)
Non-cardiovascular death	145 (13.5%)	121 (32.8%)	162 (37.1%)	1.55 (1.28-1.88)	1.61 (1.38-1.88)

Supplemental Table 5. – Cause-specific hazard ratios for major adverse cardiovascular events in patients with type 2 myocardial infarction *or* myocardial injury alone *who survive from their initial presentation to 30 days*; unadjusted and fully adjusted cox-regression models.

	Major Adverse Cardiovascular Events (MACE)	
	Unadjusted HR (95% CI)	***Adjusted HR (95% CI)***
**Age (per 10-year increase)**	1.56 (1.39-1.75)	1.53 (1.34-1.75)
**Sex (male)**	1.08 (0.84-1.38)	1.26 (0.97-1.64)
**Haemoglobin (per 10 g/L reduction)**	1.10 (1.04-1.16)	1.04 (0.99-1.10)
**eGFR (per 10 ml/min reduction)**	1.16 (1.10-1.21)	1.11 (1.05-1.17)
**Smoking**	0.86 (0.60-1.23)	1.39 (0.94-2.05)
**Diabetes Mellitus**	1.79 (1.36-2.35)	1.50 (1.12-2.01)
**Hypertension**	1.61 (1.24-2.10)	1.02 (0.76-1.36)
**Stroke**	1.54 (1.12-2.13)	1.12 (0.80-1.55)
**Peripheral Vascular Disease**	2.43 (1.68-3.50)	1.82 (1.21-2.74)
**Validation phase**	1.19 (0.92-1.53)	1.25 (0.96-1.63)
**Coronary Artery Disease**	2.21 (1.73-2.83)	1.71 (1.31-2.24)

eGFR = estimated glomerular filtration rate. Patients without coronary artery disease as referent group.

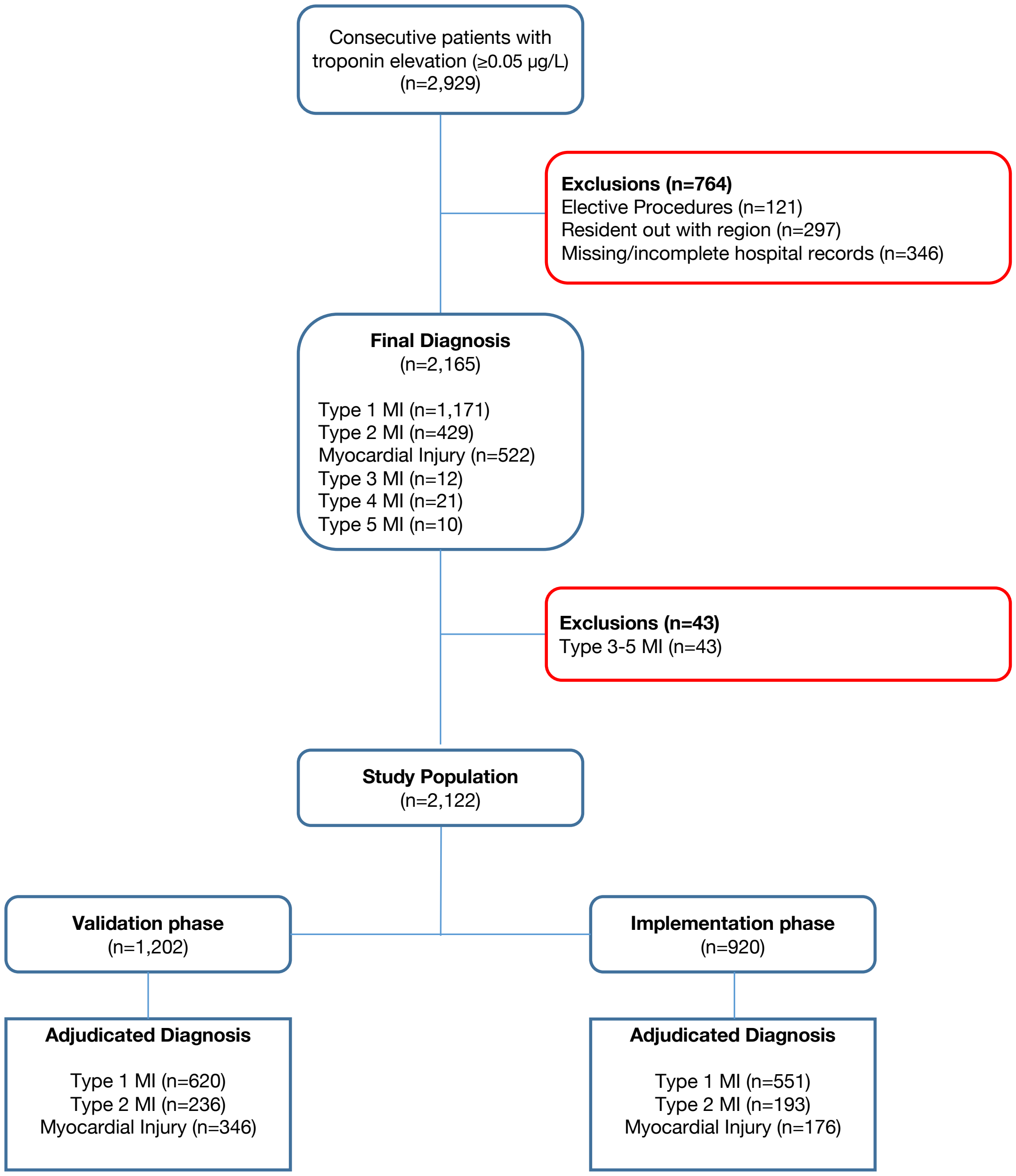

Supplemental Figure 1. – CONSORT Diagram with identification of the study population. Consecutive patients with elevation in cardiac troponin concentration were identified (≥0.05 µg/L). We excluded patients who underwent elective procedures, residents not local to our region or with missing or incomplete records. After adjudication, we excluded those with Type 3-5 myocardial infarction.

Supplemental Appendix 1. Analysis code

All analysis was performed using R (version 3.2.2) using the *survival* and *cmprsk* packages. For transparency, the analysis code is available open source via GitHub.[3]

Available at https://github.com/a-r-chapman/type_2_outcomes

Supplemental Appendix 2. Additional information on diagnostic adjudication

Criteria for adjudication of patients with myocardial necrosis

**Type 1 myocardial infarction**	Myocardial necrosis (any cardiac troponin I [cTnI] concentration above the upper reference limit) with rise and or fall in cTnI concentration where serial testing was available AND symptoms OR signs of myocardial ischaemia
**Type 2 myocardial infarction**	Myocardial necrosis (any cTnI concentration above the upper reference limit) with rise and or fall in cTnI concentration where serial testing was available AND symptoms OR signs of myocardial ischaemia AND evidence of increased oxygen demand (e.g. tachyarrhythmia, hypertrophy) or reduced supply (e.g. hypotension, hypoxia or anaemia) in context of alternative clinical diagnosis
**Myocardial injury**	Myocardial necrosis (any cTnI concentration above the upper reference limit) without symptoms OR signs of myocardial ischaemia in context of alternative clinical diagnosis

The process of adjudication was conducted by two cardiologists independently. Both had access to the electronic patient record. The adjudicated diagnosis was reached by evaluating the attending clinicians documentation of the presenting complaint, past medical history, cardiovascular risk factors and clinical examination findings including routine observations (pulse, blood pressure, pulse oximetry, temperature and conscious level). All investigation results undertaken by the attending clinician were available for review, including biochemistry and haematology results, the 12 lead electrocardiogram, echocardiogram, chest X-ray and invasive coronary angiography findings when performed. Both adjudicating cardiologists had access to the final discharge letter documenting the attending clinicians' final diagnosis. We did not apply specific criteria to define supply or demand imbalance,[1] but adjudicated myocardial supply or demand imbalance on an individual patient basis, in line with most studies in this area.[2]

Upper reference limit = 0.05 µg/L

Supplemental Appendix 3. Additional information on classification of cardiovascular death

ICD Code	Definition
**Ischaemic heart diseases**	
I20	Angina pectoris
I21	Acute myocardial infarction
I22	Subsequent myocardial infarction
I23	Certain current complications from acute myocardial infarction
I24	Other acute ischaemic heart diseases
I25	Chronic ischaemic heart disease
**Other forms of heart disease**	
I34	Non-rheumatic mitral valve disorders
I35	Non-rheumatic aortic valve disorders
I36	Non-rheumatic tricuspid valve disorders
I37	Pulmonary valve disorders
I42	Cardiomyopathy
I43	Cardiomyopathy in diseases classified elsewhere
I46	Cardiac arrest
I48	Atrial fibrillation and flutter
I49	Other cardiac arrhythmias
I50	Heart failure
I51	Complications and ill-defined descriptions of heart disease
**Cerebrovascular diseases**	
I60	Subarachnoid haemorrhage
I61	Intracerebral haemorrhage
I62	Other nontraumatic intracerebral haemorrhage
I63	Cerebral infarction
I64	Stroke, not specified as haemorrhage or infarction

I65	Occlusion and stenosis of precerebral arteries, not resulting in infarction
I66	Occlusion and stenosis of cerebral arteries, not resulting in infarction
I67	Other cerebrovascular diseases
I68	Cerebrovascular disorders in diseases classified elsewhere
I69	Sequelae of cerebrovascular disease

Supplemental References

1. Saaby L, Poulsen TS, Diederichsen AC, Hosbond S, Larsen TB, Schmidt H, Gerke O, Hallas J, Thygesen K, Mickley H. Mortality rate in type 2 myocardial infarction: observations from an unselected hospital cohort. *Am J Med*. 2014;127:295–302.

2. Sandoval Y, Thygesen K. Myocardial Infarction Type 2 and Myocardial Injury. *Clin Chem.* 2016; DOI: 10.1373/clinchem.2016.255521.

3. Chapman AR. Long term outcomes in type 2 myocardial infarction: analysis code. *GitHub repository.* 2017. Available online at https://github.com/a-r-chapman/type_2_outcomes.
